# A Comprehensive Investigation of Genomic Variants in Prostate Cancer Reveals 30 Putative Regulatory Variants

**DOI:** 10.3390/ijms24032472

**Published:** 2023-01-27

**Authors:** Mahdieh Labani, Amin Beheshti, Ahmadreza Argha, Hamid Alinejad-Rokny

**Affiliations:** 1 BioMedical Machine Learning Lab (BML), The Graduate School of Biomedical Engineering, UNSW Sydney, Sydney, NSW 2052, Australia; 2 Data Analytic Lab, Department of Computing, Macquarie University, Sydney, NSW 2109, Australia; 3 The Graduate School of Biomedical Engineering, UNSW Sydney, Sydney, NSW 2052, Australia; 4 UNSW Data Science Hub, The University of New South Wales, Sydney, NSW 2052, Australia; 5 Health Data Analytics Program, Centre for Applied AI, Macquarie University, Sydney, NSW 2109, Australia

**Keywords:** prostate cancer, somatic point mutations, copy number variation, regulatory variant, Hi-C, personalized medicine, biomedical machine learning

## Abstract

Prostate cancer (PC) is the most frequently diagnosed non-skin cancer in the world. Previous studies have shown that genomic alterations represent the most common mechanism for molecular alterations responsible for the development and progression of PC. This highlights the importance of identifying functional genomic variants for early detection in high-risk PC individuals. Great efforts have been made to identify common protein-coding genetic variations; however, the impact of non-coding variations, including regulatory genetic variants, is not well understood. Identification of these variants and the underlying target genes will be a key step in improving the detection and treatment of PC. To gain an understanding of the functional impact of genetic variants, and in particular, regulatory variants in PC, we developed an integrative pipeline (AGV) that uses whole genome/exome sequences, GWAS SNPs, chromosome conformation capture data, and ChIP-Seq signals to investigate the potential impact of genomic variants on the underlying target genes in PC. We identified 646 putative regulatory variants, of which 30 significantly altered the expression of at least one protein-coding gene. Our analysis of chromatin interactions data (Hi-C) revealed that the 30 putative regulatory variants could affect 131 coding and non-coding genes. Interestingly, our study identified the 131 protein-coding genes that are involved in disease-related pathways, including Reactome and MSigDB, for most of which targeted treatment options are currently available. Notably, our analysis revealed several non-coding RNAs, including *RP11-136K7.2* and *RAMP2-AS1*, as potential enhancer elements of the protein-coding genes *CDH12* and *EZH1*, respectively. Our results provide a comprehensive map of genomic variants in PC and reveal their potential contribution to prostate cancer progression and development.

## 1. Introduction

Prostate cancer is the second most common cancer and the fifth leading cause of cancer death among men, with almost 1.3 million new cases and 359,000 associated deaths worldwide in 2018 [1]. Genetic instability is one of the hallmarks of cancer cells. This occurs with both single point mutations and chromosomal abnormalities. However, a small number of them, called drivers, contribute to oncogenesis, while the majority are passenger mutations accumulated during cancer progression. Systematic identification of driver genes from large background noise is important. In this study, we identified putative genomic variants associated with an increased risk of cancer susceptibility from large background noise in order to provide an appropriate list of genes with a potential impact on PC progression.

Identification of cancer-associated genomic variants has focused on both protein-coding and non-coding genes. For example, Functional Analysis through Hidden Markov Models (FATHMM) [2] was used to prioritize genomic variants in the protein-coding genes. However, most of the genome is in non-coding regions, including non-coding RNAs and non-annotated regions, and the majority (>90%) of genomic variants occur in these regions [3]. Thus, determining the effect of genomic variants in non-coding regions is necessary. To this aim, there are computational tools that link genomic variants to different regulatory elements obtained from international projects, such as the Encyclopedia of DNA Elements (ENCODE), the Functional Annotation of the Mammalian Genome (FANTOM), the Roadmap Epigenomics Project, and Genotype-Tissue Expression (GTEX). For example, FunSeq2 [4] was designed to identify and prioritize non-coding somatic point mutations using various resources including ENCODE and other publications [5]. This pipeline firstly assigns a score to genomic variants based on the overlapping of these genomic variants with various genomic features, including regulatory elements (enhancer marks H3K4me1 and H3K27ac, DNA methylation), a network of genomic variants associated with genes, and recurrent elements across cancer samples (i.e., those variants identified by whole genome sequencing of at least two samples). FunSeq2 then assigns a specific weight to the features based on the 1-Shannon entropy. RegulomeDB is another tool [6] designed to prioritize disease-associated single nucleotide polymorphisms (SNPs). This method employs a heuristic scoring system that assigns a specific score to each SNP based on the number of overlaps between SNPs and an integrated regulatory database including TFBSs, chromatin states of different cell types, and eQTL data. Chen et al. [7] also developed an enrichment analysis to test whether any risk-associated SNPs are located in the functional genomic regions, including UCSC annotated coding regions (exon and snoRNA/miRNA) and regulatory regions, as well as binding regions for transcription factors (TFs), histone modifications (HMs), DNase I hypersensitivity (DHSs), and RNA Polymerase IIA (POLR2A). RegulemeDB, HAploReg, and Variant Effect Predictor (VEP) toolsets also map GWAS SNPs to regulatory elements to identify functional GWAS variants [8].

There is another category of methods using machine learning techniques to predict the potential impact of genomic variants. These methods are supervised methods, which have been trained using functional annotations to determine pathogenic variants. New genomic variants can then be classified using this information. For example, DeepSEA (deep learning-based sequence analyzer) [9] uses a convolutional neutral network (CNN)-based framework to predict the effect of chromatin factors (transcription factor binding, DNase I sensitivity, histone mark profile) on genomic sequences. In the prioritization part, DeepSEA predicts regulatory mutations using boosted logistic classifiers via eQTL data, through non-coding trait-associated SNPs identified in GWAS studies from the US National Human Genome Research Institute’s GWAS Catalog. Chengliang et al. also presented iCAGES (integrated Cancer Genome Score) [10], a statistical framework that prioritizes cancer driver mutations, genes, and targeted drugs. This method first integrates different prioritization tools (FunSeq2, SIFT, FATHMM, VEST, Mutation Taster, Phylop, PolyPhen2, GERP++, Mutation Assessor, LRT, SiPhy, and LRT) to identify candidate single point mutations and structural variations in protein-coding and non-coding regions. In the second layer, iCAGES takes the associated genes with the variations identified in the previous step, together with the gene list from the Phenolyzer tool, to assign a score for each gene based on a logistic regression model. Finally, this method links identified genes to specific drugs and calculates a specific score for each drug based on its effectiveness. Shengcheng et al. also presented SURF (Score of Unified Regulatory Features) [11] which uses features from RegulomeDB and DeepSEA tools and then applies a random forest model to predict the effect of a genomic variant (SNP) in promoter and enhancer regions.

The above-mentioned methods determine the overlapping of genomic variants in the coding and non-coding regions; however, they are not able to identify the potential impact of the variants and how these variants affect gene expression. Integrative analyses have been used previously in cancer biomarkers discovery [1,12,13,14,15,16,17,18]; however, none of these platforms integrate chromosome conformation capture data to identify the impact of regulatory variants in PC. Here, we have developed a new integrative pipeline, Associated Genomic Variants (AGV), which uses high-throughput chromosome conformation capture data (Hi-C), RNA-Seq, ChIP-Seq, and a list of genetic variants to link the variants to target genes in prostate cancer. We applied AGV to the genomic variants of 194 PC patients obtained from the International Cancer Genome Consortium (ICGC) and PC-associated GWAS SNPs from GWAS Catalog and identified the candidate coding and non-coding variants and their associated target genes.

**Figure 1:**
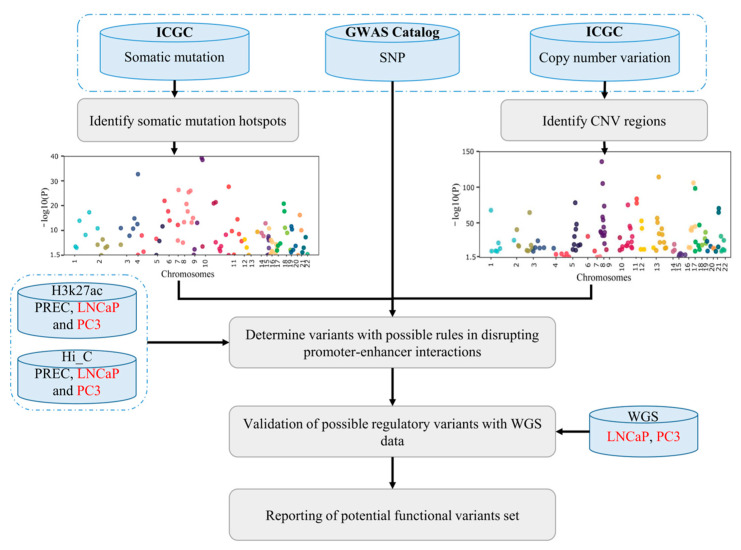
An overview of AGV. The AGV pipeline first makes a list of associated genomic variants including GWAS SNPs, somatic point mutations, and CNV regions. AGV then uses Hi-C and H3K27ac to determine variants with possible rules in disrupting promoter–enhancer interactions. Finally, AGV reports a list of functional genomic variants with a possible role in PC.

To achieve this, AGV first identifies hotspots of PC-associated somatic point mutations and CNV regions (genomic regions where CNVs are overlapping—CNVRs) and the coding and non-coding genes affected by these variants. AGV then uses H3K27ac ChIP-Seq marks to identify variants that have occurred in the enhancer regions. Using Hi-C interactions from normal and cancer cell lines, AGV generates a list of genetic variants with potential regulatory functions. Finally, we validated the PC-associated variant identified in this study using independent whole genome sequencing data from the same PC cell line. An overview of the AGV pipeline is provided in Figure 1.

The main innovations and contributions of our work are as follows:

1. This is the first study that comprehensively considers GWAS SNPs, somatic point mutations, and CNVs, while previous methods have only considered somatic mutations and GWAS SNPs to identify functional cancer-associated variants.
2. In comparison to other studies [2], which have mainly considered genomic variants in protein-coding genes, in this study we analyzed both coding and non-coding regions.
3. Most of methods that determine associated genomic variants in non-coding regions, such as FunSeq2 [4], DeepSEA [9], RegulomeDB [6], and SURF [11], are developed for general diseases, and they may not work well for a specific cancer.
4. We used an innovative strategy to identify hotspot somatic point mutation regions, which can be used in further studies to identify hotspot regions in cancer. The proposed method is built on window analysis for the detection of hotspot somatic mutation regions, which is an effective strategy for identification of hotspot regions, whereas other methods, such as FunSeq2 [2] and iCAGES [10], did not report highly mutable regions.

## 2. Results

### 2.1. Making a Comprehensive Map of Prostate Cancer-Associated Genomic Variants

**Figure 2:**
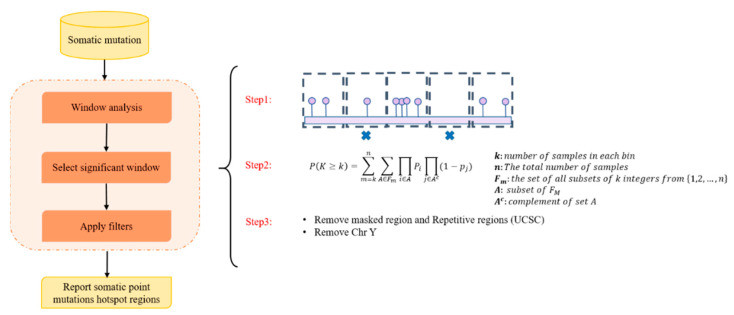
The schematic workflow used in this study to identify somatic point mutation hotspot regions. This analysis consists of three main steps: (1) window analysis, (2) selection, and (3) filtering. In the first step, the tool divides the genome into 21bp bins and then counts the number of samples with at least one SPM that overlaps with the window. In the selection step, a Poisson binomial distribution is used to select significant bins (*p*-value < 0.05). Lastly, in the filtering step, the problematic hotspot regions and chromosome Y are excluded from the final list of hotspot regions.

We first collected all prostate cancer-associated GWAS SNPs from [19], considering GWAS SNPs with (*p*-value < 5 × 10^−8^) (Supplementary Table S1a). We then used somatic point mutations from 194 ICGC PC samples (including 10,154,740 single point mutations) to identify hotspot regions. Somatic point mutations (SPMs) are distributed in the whole genome randomly, and most of them are passengers. Therefore, we considered somatic hotspot regions as the genomic regions with enrichment of somatic point mutations in the PC samples. Hotspot regions have been widely noted to be important in different cancer types [20,21]. The identification of somatic point mutations hotspot regions has three main steps including window analysis, selection of significant windows, and a filtering process. Figure 2 illustrates the framework used for the identification of hotspot regions in this study.

**Figure 3:**
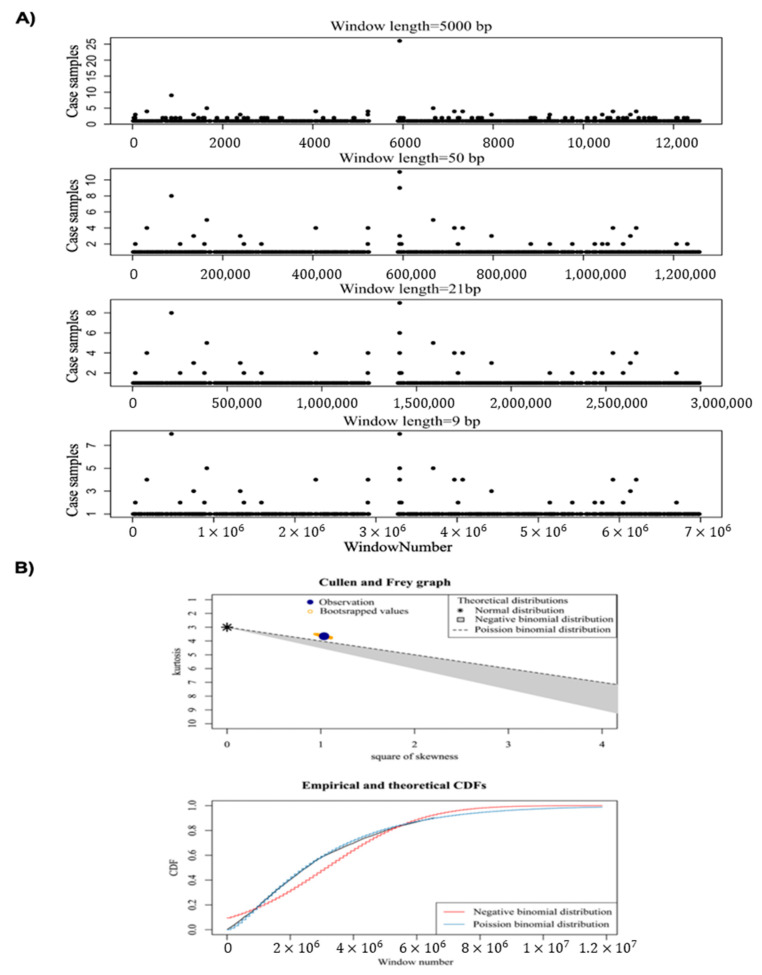
(**A**) Distribution of SPMs for different window sizes (500, 50, 21, and 9 bp) on chromosome 22. (**B**) The probability distribution for identified 21 bp bins by Cullen and Frey graph, and CDF plot.

In the first step, window analysis is used to divide the genome into windows of fixed size, and the number of samples encompassing SPM within the window is then counted. In this study, window sizes of 9, 21, 50, and 5000 bp were tested to detect the optimal window size, and the results showed that there was no significant difference in terms of the number of samples in each region between window sizes of 21 bp and 9 bp (Figure 3A). Therefore, we selected a window length of 21 bp as the optimal window size. 

In total, 21,966 windows containing at least one sample with SPM were detected. We then used Poisson binomial distribution to determine the significance of observing k samples containing somatic mutations in a 21 bp window. To determine the best-fitted distribution for the selection of statistically significant windows (*p*-value < 0.001), we used Skewness and Kurtosis and CDF (Figure 3B) (see method for more details).

**Figure 4:**
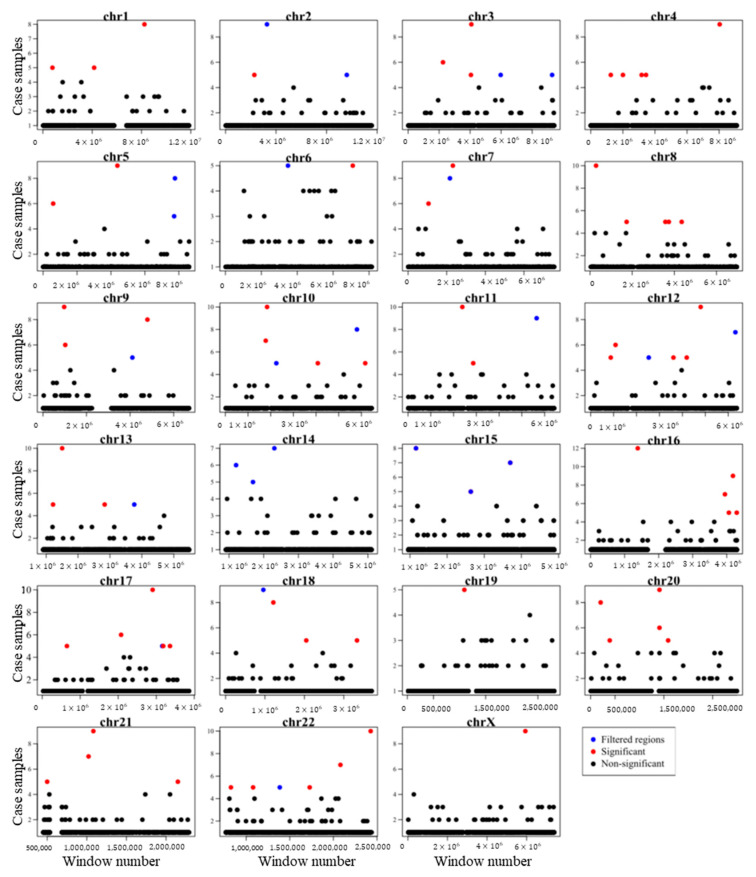
A genome-wide overview of somatic mutation hotspots (red dots) and filtered regions (containing masked regions and repetitive regions (blue dots) and non-significant regions (black dots)) was identified in this study. The figure illustrates the distribution of 21 bp bin-size windows encompassing PC-related somatic point mutations across the genome. The *x*-axis shows the window number and *y*-axis shows the number of case samples covered by the window. For each window, our proposed method calculates the *p*-value of mutation recurrence using the Poisson binomial distribution. Problematic regions, including masked regions and repetitive regions, were then excluded and bins with a *p*-value < 0.001 were selected.

As a result, we identified 71 somatic mutation hotspot regions that were significantly associated with PC (Figure 4 and Supplementary Table S1b).

To gain a comprehensive list of PC-associated variants, we also used copy number variants available for PC samples from the ICGC datasets to identify PC-associated CNVRs (genomic regions where CNVs are overlapping). To achieve this, we used the CNV maps for 11,564 CNVs (3625 deletions and 7939 duplications) of 194 patients from the ICGC [22] and publicly available data for 2392 healthy individuals from the 1000 Genomes Project [23] containing the genomic coordinates for 32,449 CNVs (22,318 deletions and 10,131 duplications). To identify PC-associated CNVs, a genome-wide genetic association analysis needs to be performed between the CNV regions and the observed phenotypes. However, one of the major obstacles in a CNV-based genome-wide association study occurs when categorizing CNVs across all cases (individuals with the phenotype of interest) and controls (healthy individuals), because CNVs are inconsistent in sequence, size, and genomic coordinates across individuals. To address this issue, one effective approach is to build CNVRs (genomic regions where CNVs are overlapping—CNVRs) prior to identifying those CNVRs that are statistically associated with the phenotype of interest. In this study, we used the PeakCNV [24] method, which can determine CNVRs that are significantly associated with PC. It considers the dependency between CNVs to remove CNVRs that overlap or co-occur with true positive CNVRs. PeakCNV uses an artificial intelligence-based technique that firstly identifies deleted and duplicated CNVRs that are significantly overrepresented among cancer samples and then identifies clusters of CNVRs that are deleted/duplicated in the samples and are proximally close to each other. PeakCNV then reports the best representative CNVR for each cluster as the candidate CNVRs. As a result, we identified 216 duplicated CNVRs and 75 deleted CNVRs that were significantly associated with PC (Supplementary Table S1c).

In total, we listed 2354 PC-associated genomic variants, including 1992 GWAS SNPs, 71 hotspot regions, and 291 CNVRs. We then went on to investigate how these variants contribute to the progress of prostate cancer.

### 2.2. Linking PC-Associated Genomic Variants to Coding and Non-Coding Genes

**Figure 5:**
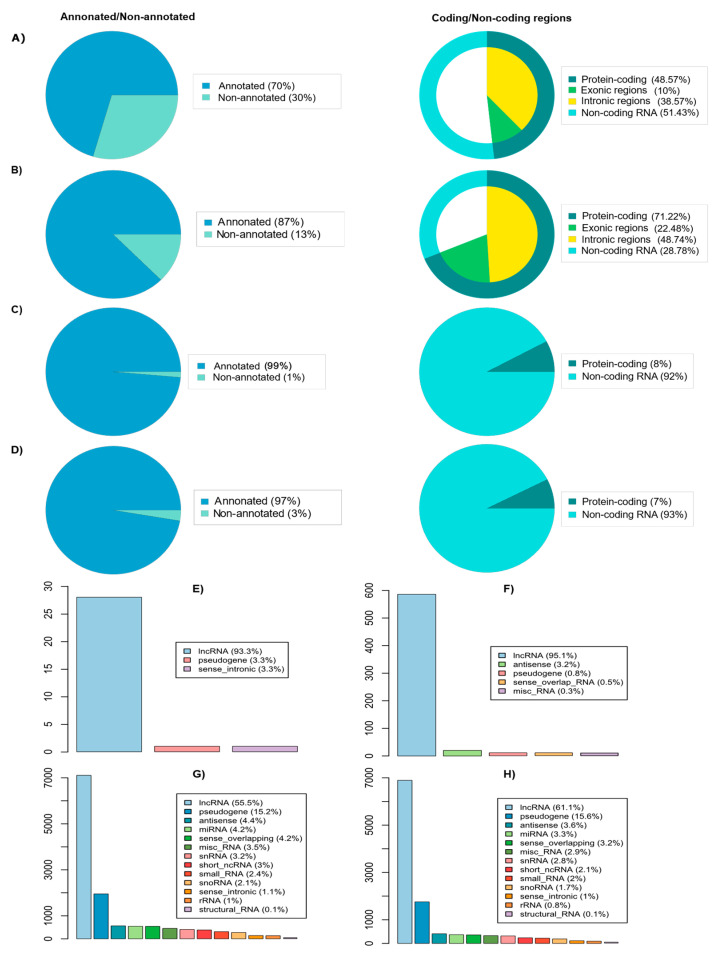
Linking genomic variants to coding and non-coding genes. (**A**) Somatic point mutation hotspots; (**B**) GWAS SNPs; (**C**) duplicated CNVRs; (**D**) deleted CNVRs. The left panel shows the percentage of genomic variants associated with the genes (annotated regions) or no genes associated with the genomic variants (non-annotated regions). The right panel shows the fraction of these genomic variants that are protein-coding or non-coding genes. The greater fraction of hotspot regions and CNVRs are located in non-coding genes, while less than 30% of GWAS SNPs are located in non-coding genes. (**E**–**H**) show the percentage of linking of different types of genomic variants, including (**E**) somatic point mutation hotspots; (**F**) GWAS SNPs; (**G**) duplicated CNVRs; (**H**) deleted CNVRs into noncoding RNA. *y*-axis represents the number of different types of RNA associating with genomic variants.

To determine genes related to PC-associated genomic variants from the analysis in the previous step, we overlapped the coordinates of the genomic variants with the human reference genome (see Section 4 for more details). Notably, we identified that a greater portion of genomic variants (70% hotspot regions, 87% GWAS SNPs, 99% of duplicated, and 97% of deleted CNVRs) were associated with coding and non-coding genes (Figure 5 and Supplementary Table S2). Interestingly, we observed that a greater fraction of hotspot regions and CNVRs is in non-coding genes, while a greater portion of GWAS SNPs is in protein-coding genes. We also explored the distribution of genes associated with genomic variants in non-coding RNAs and found that more than 50% of these non-coding genes are lncRNAs (Figure 5).

### 2.3. Identify Variants with Likely Regulatory Function

**Figure 6:**
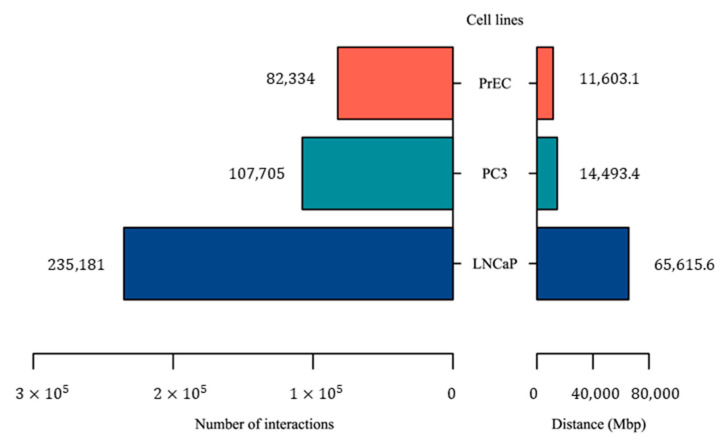
The number and distance of statistically significant Hi-C interactions in cancer cell lines (PC3 and LNCaP) and healthy cell line (PrEC).

Of the 2,354 PC-associated genomic variants identified in this study, 1,026 of them are located in non-coding regions, particularly in non-coding RNAs. However, the majority of these non-coding variants are of unknown function. Here, we hypothesize that some of these variants may have a regulatory function. To identify these regulatory variants, we first used Hi-C interactions and H3K27ac Chip-Seq signals to identify enhancer–promoter interactions. We used Hi-C interactions from two prostate cancer cell lines (PC3 and LNCaP) and one healthy cell line (PrEC). HiC-Pro [25] was used for mapping, trimming and valid interaction calling. MaxHiC [26] and MHiC [27] were used to identify statistically significant interactions (*p*-value < 0.001). As a result, 107,705, 235,181, and 82,334 significant Hi-C interactions were identified in PC3, LNCaP, and PrEC cell lines, respectively. The number of significant Hi-C interactions and their distance were higher in both prostate cancer cell lines compared to the normal PrEC cell line (Figure 6 and Supplementary Table S3), which indicates that Hi-C interactions in normal cells were often subdivided into multiple smaller interactions in cancer cells.

H3K27ac signals were then used to identify enhancer marks. We considered those Hi-C interactions where one side of the interactions overlapped with H3K27ac signals as an enhancer mark and another side overlapped with promoter region of protein-coding genes, resulting in the identification of enhancer–promoter interactions (EPIs). We identified 12,266, 3653, and 3690 EPIs in LNCaP, PC3, and PrEC cell lines, respectively (Supplementary Table S4). 1130 and 3593 EPIs were only observed in PC3 or LNCaP cell lines, respectively, and not in the healthy cell line (PrEC). We then focused on these EPIs and cross-referenced them with PC-associated genomic variants to identify regulatory variants with potential functional impact in PC. We only considered those variants that overlapped with the enhancer side of the interaction. As a result, 135 SNPs, 14 hotspot regions, and 213 duplicated and deleted CNVRs were overlapped with EPIs in the LNCaP cell line. We also identified 51 SNPs, 7 hotspot regions, and 226 duplicated and deleted CNVRs that overlapped with EPIs in the PC3 cell line (Supplementary Table S5). Of particular interest, we identified a GWAS SNP rs10993994 that overlapped with EPI chr10:5130000-51535000;chr10:51580000-51585000 A study on this GWAS SNP by Bicak et al. [28] showed that it has a regulatory function for MSMB genes [28].

The 646 potential regulatory variants interacted with 13,858 protein-coding genes. Interestingly, 278 of these variants are located in the body of non-coding RNAs, mostly lncRNAs. For example, lncRNA *HOTAIR* encompassed two regulatory variants. This lncRNA was previously identified as an enhancer RNA that regulates the protein-coding gene *MDM2*, and this has been validated by different integrative meta-analyses [29,30].

We then used whole genome sequencing (WGS) of prostate cancer cell lines (PC3 and LNCaP) to see how many of the regulatory variants we used in this study were replicated in whole genome sequencing of the same cancer cell line. As a result, 23 GWAS SNPs, 2 hotspot regions, and 93 duplicated and deleted CNVRs that overlapped with EPIs in the LNCaP cell line were also replicated in the WGS data and 2 GWAS SNPs, 1 hotspot region, and 67 duplicated and deleted CNVRs were also replicated in the PC3 cell line (see Supplementary Table S6 for more details).

**Figure 7:**
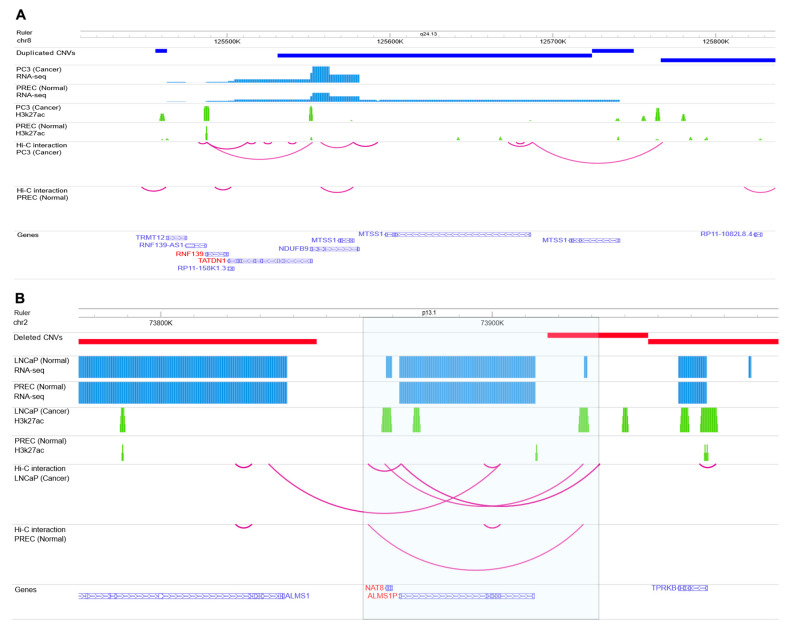
(**A**) Example of a regulatory variant in cancer cell line PC3. The figure demonstrates the RNA-Seq, H3K27ac signals, and Hi-C chromatin interactions map of normal (PREC) and prostate cancer (PC3) cells on Chromosome 8. The highlighted box shows one of the PC-associated CNVRs identified in this study that was also observed in PC3 WGS. There is an EPI in the cancer cell line (the EPI was not observed in the healthy cell line) where the enhancer side of the interaction overlapped with CNVR. Interestingly, the left side of this interaction is promoter regions of *RNF139* and *TADN1*, and the right side (enhancer region) also has an active H3K27ac signal. The expression of *NDUFB9* is much higher in the cancer cell line compared to the healthy cell line. (**B**) Example of the regulatory variant in cancer cell line LNCaP. The figure demonstrates the RNA-seq, H3K27ac signals, and Hi-C chromatin interactions map of normal (PREC) and prostate cancer (LNCaP) cells on Chromosome 2. The highlighted box shows one of the PC-associated CNVRs identified in this study that was also observed in LNCaP WGS. There is an EPI in the cancer cell line (the EPI was not observed in the healthy cell line) where the enhancer side of the interaction overlapped with CNVR. Interestingly, the left side of this interaction is promoter regions *NAT8* and *ALMS1P* genes, and the right side (enhancer region) also has an active H3K27ac signal. WashU Epigenome Browser has been used to generate the figure.

For example, CNVR (chr8:127394134-127501076) overlapped with the enhancer side of EPI, whereas the other side overlapped with the protein-coding genes *TATDN1* and *RNF139*. More importantly, both H3K27ac and RNA-seq data showed a much higher signal in the cancer cell line compared to the healthy cell line (Figure 7A), indicating a possible effect of this PC-associated duplicated CNVR in enhancing the expression of *NDUFB9* and *MTSS1* genes in prostate cancer. Interestingly, *MTSS1* has been reported as the metastasis driver gene in a subset of human melanomas [31].

Deleted CNVR (chr2:73916673-73947014) is another example of the PC-associated regulatory variants identified in this study that was also observed in the whole genome sequence of the prostate cancer cell line. As Figure 7B shows, there is a Hi-C interaction in the PC3 cancer cell line in which one side of the interaction overlapped with the potential enhancer region and another side overlapped with *NAT8* and *ALMS1P*. The expression of these genes was significantly increased in the cancer cell line, indicating that this CNVR may act as the potential genomic variant disrupting this enhancer–promoter interaction. Based on the literature search, *ALMS1P* is one of the causative genes identified for various diseases, while its physiological function and pathological significance in different diseases are still unknown [32].

We next performed a pathway analysis on the genes associated with the identified regulatory regions. We used ShinyGO [33] to determine genes that were enriched in disease-related pathways. To achieve this, we first used a complete list of pathway databases in ShinyGO to assess the relative biological importance of the identified regulatory genes (see methods for more details). We then mapped the regulatory genes to curated gene sets/pathways to screen for involvement in known cancer and other molecular processes.

Our analysis showed that ~44% of interacting genes are associated with previously known curated gene sets/pathways (cutoff of *p*-value < 0.05). The most highlighted gene set is LASTOWSKA_NEUROBLASTOMA_COPY_NUMBER_DN database from the msigdb [34] database, which contains genes with copy number losses in primary neuroblastoma tumors. These deleted copy number variations are the major cause of gene transcription. We identified 17% of interacting genes that were involved in this pathway (9.55-fold change, *p*-value < 7.49 × 10^−13^). Furthermore, 15 genes were expressed in the CUX1-19635798-MULTIPLE HUMAN CANCER CELL TYPES-HUMAN transcription factor binding site profile database [35], which contains 2406 expressed genes with transcription factor binding evidence in multiple human cancer cell types (Supplementary Table S7).

Intriguingly, we also identified two other cancer-associated pathways including WOO_LIVER_CANCER_RECURRENCE_DN [18] and VANTVEER BREAST CANCER ESR1 UP [36]. Some of the genes in these pathways include *ALAS1, ACAA1,* and *ACOX2* which are negatively correlated with recurrence-free survival in patients with hepatitis B-related (HBV) hepatocellular carcinoma (HCC). Interestingly, it has been shown that chronic hepatitis B virus (HBV) infection is a leading cause of hepatocellular carcinoma (HCC) [37].

**Figure 8:**
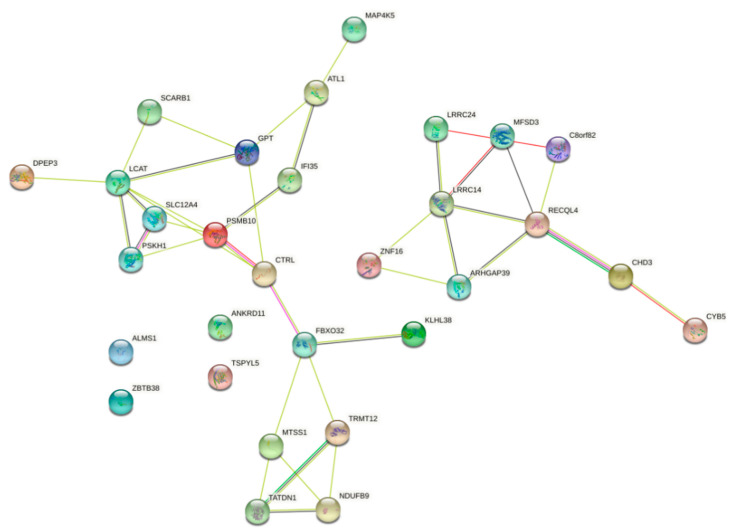
Enriched proteins in the KEGG pathway for cancer.

We then used the STRING-db website [38] to retrieve the protein–protein interactions for the interacting protein-coding genes. This network provides insight into which proteins are associated with other proteins, and the development of new molecular drugs that control the interactions between causal proteins interactions may be beneficial for disease therapy. Figure 8 illustrates the protein networks for the top 30 enriched genes in the prostate cancer-related KEGG pathway. This analysis provides a list of the most significant target proteins with a cutoff *p*-value < 0.05. For example, our analysis finds that zinc finger protein 16 (*ZNF16*), which has been shown to have a potential role in DNA damage, and Cisplatin (used as an anticancer drug) prevent the overexpression of this protein [39]. Furthermore, RecQL4 was reported as a novel molecular target for cancer therapy in 2021 [40], with a prognostic role in metastatic tumor samples [41].

## 3. Conclusions

In this study, we have developed a new pipeline, AGV, to systematically detect putative regulatory variants, including copy number variations, SNPs, and hotspot somatic mutations, for prostate cancer. The AGV pipeline can be easily integrated into any other pipeline; thus, it is useful for downstream analysis of any disease. AGV consists of three main steps that can be run independently based on the user request. Firstly, it generates a list of hotspot somatic mutations, CNVRs, and GWAS SNPs, together with their associated coding and non-coding genes. To determine hotspot somatic mutation regions, AGV employs a sliding window algorithm that splits the human genome into fixed size windows and then computes the significant windows. AGV then uses an AI-based algorithm (PeakCNV) to generate a list of true positive CNVRs. The identified genomic variants will then be integrated with Hi-C data and H3K27ac signals to provide a list of potential functional EPIs. We identified 30 regulatory variants that potentially disrupt enhancer–promoter interactions in the PC-related cancer cell line. The regions that encompass these variants, interact with 131 genes where each gene can be targeted by multiple regulatory variants.

The development of innovative deep learning algorithms, which have proven to outperform traditional approaches in genomics, transcriptomics, and clinical biomarker identification [42,43,44], can be used in integration with these methods to provide a better understanding of the mechanisms that underlie cancers.

## 4. Materials and Methods

### 4.1. GWAS Dataset

GWAS SNPs were downloaded from the GWAS Catalog (https://www.ebi.ac.uk/gwas/docs/file-downloads—accessed on 14 June 2020) and GWASdb v2 (http://jjwanglab.org/gwasdb—accessed on 15 June 2020). We considered only those SNPs that were associated with prostate cancer. All GWAS SNPs with *p*-value < $10^{-8}$ were excluded from the analysis.

### 4.2. Somatic Point Mutations Dataset

The genomic coordinates of somatic point mutation (SPM) for prostate cancer were obtained from the International Cancer Genome Consortium (ICGC) [45]. In total, there were 10,154,740 SPMs from 1037 PC patients across six projects (PRAD-US, PRAD-CA, PRAD-UK, EOPC-DE, PRAD-CN, and PRAD-FR) from the United States, the United Kingdom, Canada, Germany, China, and France.

### 4.3. Identification of Somatic Point Mutation Hotspots

To identify somatic point mutation hotspots, our pipeline firstly counted the mutation recurrence for fixed bin size regions (bin length = 21 bp). The user is able to set the window length based on the desired minimum recurrence frequency. The *p*-value of mutation recurrence was computed using a Poisson binomial distribution model to determine the significance of observing k samples containing somatic mutations in a 21 bp window. A Skewness and Kurtosis graph and a CDF plot were executed by “fitdistrplus” in R package. Next, the problematic hotspot regions, such as masked regions (regions with mappability score < 1 in the ENCODE 75mers alignability track in the UCSC genome browser) and repetitive regions (RepeatMasker track and simpleRepeat tracks in the UCSC genome browser) [46] were excluded. We also excluded chromosome Y in our analysis.

### 4.4. PeakCNV

To determine CNV regions (genomic regions where CNVs are overlapping—CNVRs) that are associated with disease, we proposed an AI-based method called PeakCNV, which is an extension of the SNATCNV toolset [47].

PeakCNV selects CNVRs with the lowest confounding with true positive CNVRs. To this aim, PeakCNV has three main steps, including CNVR map building, a clustering process, and a selection process. In the first step, deletion and duplication CNVR maps are built for case and control, independently; then, CNVRs that are significantly represented in cases over controls at nucleotide base are selected. In the next step, significant CNVRs are grouped into different clusters based on the similar association of CNVRs with the phenotype of interest. To achieve this, we used the DBSCAN clustering algorithm with two input features, including CNVR uniqueness (the number of case samples covered by a given CNVR after subtracting the common case samples between each pair of CNVRs), and the genomic distance between CNVRs. Lastly, it selects the most independent CNVRs from each cluster using a novel score IR-score. Independent CNVRs are those detected in the greatest number of cases and having a minimum co-occurrence with other CNVRs. PeakCNV runs with the default parameters (*p*-value < 0.05).

### 4.5. Reference Gene Annotations

FANTOM5 [48], Ensembl [49], and GENCODE [50] gene annotation files were used to curate a comprehensive reference gene list. The FANTOM5 gene annotation file was used as the backbone of our reference gene list, but when the gene annotation was absent from FANTOM5, these were acquired from Ensembl and GENCODE. The final reference gene list contained 82,539 genes, including 58,000, 24,501, and 38 genes from FANTOM5, Ensembl, and GENCODE, respectively. The genomic coordinates for CNVRs, somatic point mutations, GWAS SNPs, and gene annotations were in the hg19 genome assembly.

### 4.6. Identification of Genomic Variants Affecting Coding and Non-Coding Genes

This analysis is performed to indicate which genes are affected by the observed genomic variants in prostate cancer, including GWAS SNP (1,992 SNPs), hotspot regions (71 regions), and CNVRs (duplication: 216 CNVRs, deletion: 75 CNVRs). Bedtools v2.30.0 [51] was used to identify the overlapping between the genomic coordinates of genomic variants and genes [51]. The risk SNPs, hotspot regions, and CNVRs that were used for this analysis are provided in Supplementary Table S1. The list of genes affected by the different types of genomic variants is also provided in Supplementary Table S2.

### 4.7. Preparation of Hi-C Libraries

Hi-C data from normal human prostate epithelial cells (PrEC) and prostate cancer cell lines PC3 and LNCaP with GEO GSE73785 were downloaded using the KARAJ toolset [52] from previously published data [53]. We used KARAJ [52] to download datasets and supplementary files. Two replicates were available for each cell line. We used HiC-Pro v2.11 [25] and HiCcompare [54] with the default parameters for analyzing and aligning Hi-C data in 5 kb fragment size. We then used MaxHiC [26] and MHiC to identify statistically significant cis interactions. Here, we only considered those significant cis interactions with a *p*-value < 0.01, a read-count ≥ 10, and a distance between the two sides of the interaction of more than 5k and less than 10M. We then used our genes list to annotate Hi-C interactions with coding and non-coding genes. At least 10% overlap between gene and Hi-C fragments was considered to annotate Hi-C fragments with genes. Two replicates of each Hi-C cell line were merged to enhance the statistical power (Supplementary Table S3).

### 4.8. Identification of H3K27ac ChIP-Seq Peak Regions

H3K27ac ChIP-Seq FASTQ files for PC3, LNCaP, and PrEC cell lines were downloaded from GEO GSE57498, GSE73785, and GSE57498, respectively [53,55]. Bowtie2 [56] was then used to map the FASTQ file to the hg19 human reference genome. Peaks were then called using Model-based Analysis of ChIP-Seq (MACS2) [57] with the p−value<0.001 (Supplementary Table S9).

### 4.9. Literature Search Strategy

Our literature searches were focused on human and mouse English-language papers available in PubMed, Scopus, and Web of Science. We used data and text mining techniques to extract additional related studies [58,59,60,61,62,63,64,65,66,67,68,69,70,71,72,73]. A knowledge-based filtering system technique was also used to categorize the texts from the literature search [74,75,76,77,78,79]. The search terms included “cancer”, “prostate cancer”, “noncoding RNA”, “enhancer”, “CNV”, “mutation”, and “copy number variations”.

### 4.10. Whole Genome Sequencing Data Processing

#### 4.10.1. Mapping of FASTQ Reads of Prostate Cell Lines to Reference Genome

We obtained WGS data for LNCaP (ATCC CRL-1740) and PC3 (ATCC CRL-1435) from published work [17] using the KARAJ pipeline. The quality checking of FASTQ files was performed using FastQC v0.11.9 [80]. Trimmomatic v0.40 [81] was then used to filter poor quality reads and trim poor quality bases (phred score < 30) from our samples. BWA-MEM v0.7.17 (r1188) [82] was then used to map sequencing reads to the human reference genome (hg19) and a sorted BAM file was generated by SAMtools v1.12 [83].

#### 4.10.2. Variant Calling

To call single nucleotide polymorphisms (SNP) and short indels from the bam files, SAMtools v1.12 mpileup and BCFtools [84] were used to interrogate indexed BAM files of reads aligned to the reference genome and generate a VCF (Variant Call Format) file of SNPs and short indel variants. Variant files (VCF) were then filtered using BCFtools with the following parameters: QUAL ≤ 30 && DP ≤ 10; where QUAL denotes minimum variance confidence and DP total depth threshold. The Control-FREEC v11.6 pipeline [85] was also used to call copy number variations from the sorted BAM files and generate duplicated and deleted variants.

### 4.11. Data Visualization

To visualize the impact of regulatory variants in Hi-C interaction and gene expression, the Washu Epigenome Browser [86] was used. In this analysis the Hi-C interactions, in conjunction with gene expression, ChIP-Seq, and genomic variants data, were used.

### 4.12. Pathway Analysis

To validate the capability of AGV in identifying meaningful genes, we used ShinyGO v0.4 [33]. It contains 72,394 gene sets for the human genome, including KEGG [87], MSigDB [88], GeneSetDB [89], and REACTOME [90]. It also has access to STRING-db [91] for the retrieval of protein–protein interaction networks. We analyzed a set of 131 genes (Supplementary Table S6) which were identified as the potential regulatory genes in our analysis. This gene list is mapped to all human gene sets in ShinyGo for enrichment analysis. ShinyGo uses a hypergeometric distribution over-representation test to calculate the *p*-value for gene set overlaps. We ran ShinyGo with the default values (*p*-value cutoff=0.05) (Supplemental Table S8).

## Data Availability

All data are publicly available and cited in the paper. The source code is available at https://github.com/mahdieh1/ProstateCancer (accessed on 26 August 2022).

## Supplementary Materials

The following supporting information can be downloaded at: https://www.mdpi.com/article/10.3390/ijms24032472/s1, Supplementary Table S1: List of genomic variants for PC that were used in this study. (a) GWAS SNPs, (b) hotspot somatic mutations, (c) CNVRs. Supplementary Table S2: List of genomic variants affecting genes. (a) SNPgenes, (b) somatic genes, (c) CNVgenes. Supplementary Table S3: List of statistically significant Hi-C interactions used in this study. (a) LNCaP cell line, (b) PC3 cell line, (c) PrEC cell line. Supplementary Table S4: List of identified enhancer–promoter interactions from Hi-C interactions for PC. (a) LNCaP cell line, (b) PC3 cell line. Supplementary Table S5: List of potential regulatory variants in (a) LNCaP cell line, (b) PC3 cell line. Supplementary Table S6: List of determined regulatory variants in (a) LNCaP cell line, (b) PC3 cell line. Supplementary Table S7: List of associated genes in regulatory variants. Supplementary Table S8: List of enriched genes with the pathway analysis. Supplementary Table S9: List of H3K27ac ChIP-seq peak regions used in this study. (a) LNCaP cell line, (b) PC3 cell line, (c) PrEC cell line. Supplementary Table S10: Definition of promoter regions that we used in our analysis.
